# Comparative safety analysis of Riluzole, Edaravone and Tofersen in ALS management: insights from FAERS database

**DOI:** 10.3389/fphar.2025.1687698

**Published:** 2025-11-12

**Authors:** Shuang Guan, Sicun Wang, Yinli Shi, Yuanyuan Leng, Yu Ming, Zhiyong Hou, Yanan Yu, Zhong Wang, Jun Liu

**Affiliations:** Institute of Basic Research in Clinical Medicine, China Academy of Chinese Medical Sciences, Beijing, China

**Keywords:** Riluzole, Edaravone, Tofersen, adverse drug events, FAERS database, Disproportionality analysis

## Abstract

**Background:**

Amyotrophic lateral sclerosis (ALS) is a progressive neurodegenerative disorder. Riluzole, Edaravone, and Tofersen, three promising treatments, have distinct profiles that merit comparative analysis to guide clinical decision-making.

**Methods:**

This study utilizes a pharmacovigilance analysis of adverse events reported in the FDA Adverse Event Reporting System database from Q1 2004 to Q2 2024. Employing disproportionality, we assessed and compared the AE signals associated with Riluzole, Edaravone, and Tofersen to elucidate their safety profiles in ALS treatment. Finally, applying the Random Walk with Restart (RWR) algorithm to the protein-protein interaction (PPI) network for selecting drug target genes that have a strong correlation genes associated with severe adverse reactions. Finally, their interactions with the target were assessed through molecular docking and transcriptome analysis.

**Results:**

The analysis included 2106 AE reports for Riluzole, 2466 AE reports for Edaravone, and 136 for Tofersen. Highlights the higher incidence of adverse reactions associated with Riluzole, including abdominal discomfort, hypoaesthesia oral, and hepatic enzyme increased, as well as a significant correlation between Edaravone and falls, gait disturbance, and aphasia. Tofersen exhibits different adverse reactions compared to Riluzole and Edaravone, such as headaches, csf red blood cell count positive. Comparative analysis revealed that the three drugs shared a serious adverse reaction, which is thrombosis. RWR analysis identified seven targets related to thrombosis caused by the three drugs, including F10 and MMP9. Subsequently, molecular docking and transcriptome analysis indicate a favorable binding interaction between the drug candidate and the F10 molecule.

**Conclusion:**

This comprehensive evaluation underscores the importance of understanding the distinct AE profiles of Riluzole, Edaravone, and Tofersen in clinical practice, providing valuable insights for personalized ALS management. Future research with rigorous prospective designs is recommended to validate these findings and explore the mechanisms underlying the reported adverse events.

## 1 Introduction

Amyotrophic lateral sclerosis (ALS), commonly known as Lou Gehrig’s disease, is a progressive neurodegenerative disorder characterized by the degeneration of both upper and lower motor neurons (Feldman et al., 2022). The clinical presentation included muscle weakness, atrophy, and ultimately paralysis, with most patients succumbing to respiratory failure within 3–5 years of symptom onset (Hardiman et al., 2017). The disease is relatively rare, with an incidence of approximately 1–2.6 cases per 100,000 people annually in the United States (Xu et al., 2019). While a small percentage of cases are familial, the majority are sporadic (Chio et al., 2011; Mead et al., 2022), and the exact etiology remains largely unknown, which complicates treatment efforts.

Treatment options for ALS are limited (Goutman et al., 2022). The only FDA-approved drug for ALS is riluzole, which has been shown to modestly extend survival by about 2–3 months and reduce the progression of symptoms. Riluzole works by inhibiting the release of glutamate, a neurotransmitter that can be toxic in excessive amounts, thereby reducing excitotoxicity in motor neurons (Doble, 1996). In 2017, Edaravone was approved as a second treatment option, which acts as a neuroprotective agent and has been shown to slow the decline in physical function in some patients (Santos and Carvalho, 2024). Tofersen is an antisense oligonucleotide used for the treatment of ALS. In 2023, Tofersen was approved in the United States for the treatment of adult amyotrophic lateral sclerosis with mutations in the superoxide dismutase 1 (SOD1) gene (Blair, 2023).

These drugs may cause adverse events (AEs) such as gastrointestinal reactions, hepatic injury, and thrombosis, which can worsen patients’ conditions in severe cases. Therefore, a thorough analysis of their safety profiles is crucial to minimize excessive ALS-type preconditioning (Bensimon and Doble, 2004; Shang et al., 2025). For instance, a related research conducted a retrospective study of 92 ALS patients treated with Riluzole, finding that 20 cases (21.7%) required treatment discontinuation due to AEs. The most common cause was elevated liver enzymes (5 cases, 5.4%), followed by interstitial pneumonitis, nausea, dizziness, and other symptoms. All AEs occurred within the first 6 months of treatment and improved after discontinuation. Three patients with interstitial pneumonitis required steroid treatment due to severe respiratory failure. This highlights the need for close monitoring of liver and lung function during the first 6 months of riluzole therapy (Inoue-Shibui et al., 2019).

The FDA’s Adverse Event Reporting System (FAERS) is a key tool for monitoring drug safety after it hits the market. It collects reports of adverse events from various sources globally. Apparent excess reporting of ALS-like conditions has been identified from analysis of both European databases and a US patient-targeted pharmacovigilance effort, as well as an early FDA data mining study (Edwards et al., 2007; Golomb et al., 2009; Colman et al., 2008). Our study hypothesizes that analyzing AEs of Riluzole, Edaravone, and Tofersen can help identify and reduce excessive ALS-type preconditioning, particularly in high-risk populations. By comparing the frequency and severity of AEs, we aim to provide more precise treatment recommendations and assist clinicians in tailoring the safest ALS treatment regimens. Therefore, this study leveraged the real-world AE data in the FAERS database to compare safety aspects, analyze the signals of adverse drug reaction (ADR) disproportionality analysis for three drugs (Riluzole, Edaravone, and Tofersen), and explore unknown or potential signals. This study aims to provide a reference for rational clinical drug use.

## 2 Methods

### 2.1 Data source

Data on post-market adverse drug reactions (ADRs) are compiled by the FAERS database, which has been openly available since 2004 and is updated every 3 months. Comprising patient demographic and administrative details (DEMO), housing drug-specific information (DRUG), coded representations of reported adverse events (REAC), reflecting patient outcomes (OUTC), indicating sources of reports (RPSR), documenting therapy initiation and cessation dates for reported drugs (THER), and outlining indications for drug administration (INDI), are the seven separate files that make up the FAERS dataset. “Riluzole,” “Edaravone,” and “Tofersen” were used as keywords in a thorough search to find all related adverse event reports from Q1 2004 to Q2 2024. The system organ class (SOC) and preferred terms (PTs) were then obtained by using MedDRA 24.0 to rectify PT names in the FAERS database.

### 2.2 Data processing

Utilizing the table function in R software, we calculated the following: a is the number of target adverse events reported by the target drug. b is the number of other adverse events reported by the target drug. c is the number of target adverse events reported by other drugs. d is the number of other adverse events reported by other drugs. Values for a, b, c, and d were derived from the REACTION file. Additionally, we collected clinical characteristics of patients experiencing adverse events related to the three drugs, including sex, age, reporting area, reporter, reporting time, and outcomes. Severe adverse outcomes in patients were defined as hospitalization, disability, life-threatening conditions, or death. This outcome data was sourced from the Outcome file corresponding to the PRIMARYID.

### 2.3 Signal mining method

The reporting odds ratio (ROR), the Medicines Healthcare products Regulatory Agency (MHRA), the Bayesian Confidence Propagation Neural Network (BCPNN), and the Multi-item Gamma Poisson Shrinker (MGPS) method were the four methods utilized for ADE signal mining (Zou et al., 2023; Li et al., 2023; Pan et al., 2024). Based on its great sensitivity and low bias, the ROR approach is widely used. To assure a minimum case mix, the MHRA approach, which is an extension of the proportional reporting ratio (PRR), combines PRR values, absolute report numbers, and chi-square values. The BCPNN method, a robust signal detection methodology that is utilized worldwide, excels in early signal recognition even with sparse or missing data; as the number of reports increases, its reliability increases. A drug-related adverse event signal was considered positive in this study if it satisfied the requirements of at least one of the four algorithms. The likelihood of false positives was reduced when all four algorithms produced a positive signal, indicating a significant relationship. Tables 1 and 2 provide the formulas for the four methods as well as the criteria for detecting signals.

**TABLE 1:** Algorithms based on four grid table.

Characteristic	Drug-related ADEs	Non-drug-related ADEs	Total
Drug	a	b	a+b
Non-drug	c	d	c+d
Total	a+c	b+d	N = a+b+c+d

a, number of reports containing both the target drug and target adverse drug reaction; b, number of reports containing other adverse drug reaction of the target drug; c, number of reports containing the target adverse drug reaction of other drugs; d, number of reports containing other drugs and other adverse drug reactions.

**TABLE 2:** Four major algorithms used for signal detection.

Algorithms	Equation	Criteria
ROR	ROR = ad/bc	lower limit of 95% CI > 1, N > 3
	SE (ln ROR)=(1/a+1/b+1/c+1/d)^0.5	
	95%CI = e^ln(ROR)±1.96(1/a+1/b+1/c+1/d)^0.5^	
PRR	PRR = a(c+d)/c/(a+b)	PRR>2, χ^2>^4, N > 3
	χ^2^ = [(ad-bc)^2](a+b+c+d)/[(a+b)(c+d)(a+c)(b+d)]	
BCPNN	IC = log_2_[a(a+b+c+d)/(a+c)(a+b)]	IC025 > 0
	E(IC) = log_2_[(a+γij)(a+b+c+d+α)(a+b+c+d+β)]/[(a+b+c+d+γ)(a+b+αi)(a+c+βj)]	
	γ = γij(a+b+c+d+α)(a+b+c+d+β)	
	95%CI = E(IC) ± 2V(IC)^0.5	
MGPS	EBGM = a(a+b+c+d)/(a+c)/(a+b)	EBGM05 > 2
	95%CI = e^ln(EBGM)±1.96(1/a+1/b+1/c+1/d)^0.5^	

95% CI, 95% confidence interval; N, the number of reports; χ^2^, chi-squared; IC, information component; IC025, the lower limit of 95% CI, of the IC; E(IC), the IC, expectations; EBGM, empirical Bayesian geometric mean; EBGM05, the lower limit of 95% CI, of EBGM.

R-Studio (version 4.3.1) and Microsoft Excel 2023 were utilized for statistical analyses and data visualizations.

### 2.4 Network toxicology analysis

#### 2.4.1 Collection of drug and disease targets and PPI network construction

The molecular structures and SMILES representations of the three drugs were retrieved from the Drugbank database (https://go.drugbank.com/). Potential drug targets were identified using the ChEMBL and SwissTargetPrediction databases, with the search scope limited to “*Homo sapiens*” to ensure biological relevance. To enhance the comprehensiveness of the results, the UniProt database (https://www.uniprot.org/) was used for cross-referencing and standardization of predictions. Targets from different sources were integrated, deduplicated, and validated for structural consistency, ultimately forming a comprehensive drug target library. Using the names of severe adverse reactions as keywords, we collected relevant validated genes from the OMIM database and HPO database to form a disease gene library.

#### 2.4.2 Network analysis and RWR algorithm

Import the adverse reactions and drug-related targets into the STRING database to construct a protein-protein interaction (PPI) network. Set the minimum required interaction score to 0.700 and remove the isolated nodes from the network. Then, import the PPI results into Cytoscape 3.9.0 software and use the “MCC” algorithm to screen and obtain the top 10 adverse reaction-related targets (Chin et al., 2014). Merge the two PPI networks and use the screened targets as seed nodes to perform the RWR algorithm. RWR is a graph-based algorithm used for ranking nodes in a network. It operates by simulating random walks on the graph, with a “reset” probability that allows the walker to return to the starting node, also known as the seed node, at each step. This reset probability can be considered a measure of the importance of the starting node (Kohler et al., 2008). The R package dnet (Fang and Gough, 2014) is used to conduct RWR analysis to identify the top 10 nodes with the highest affinity scores in PPI networks.

#### 2.4.3 Molecular docking and transcriptome analysis validation

The 3D structure of the core target protein (in PDB format) was downloaded from the Protein Data Bank (PDB; https://www.rcsb.org/), while the structural data of small molecule drugs (in SDF format) were obtained from the PubChem database (https://pubchem.ncbi.nlm.nih.gov/). Molecular docking simulations were conducted using the online-based molecular docking tool CB-Dock2 (Liu et al., 2022). The ligand molecule can spontaneously bind to the receptor protein when its binding energy is less than 0, and a lower binding energy indicates a tighter binding between the two. The transcriptome data of ALS patients were retrieved from the GEO database to verify the core target.

## 3 Result

### 3.1 Descriptive analysis

#### 3.1.1 Comprehensive analysis of adverse event reporting statistics

**FIGURE 1:**
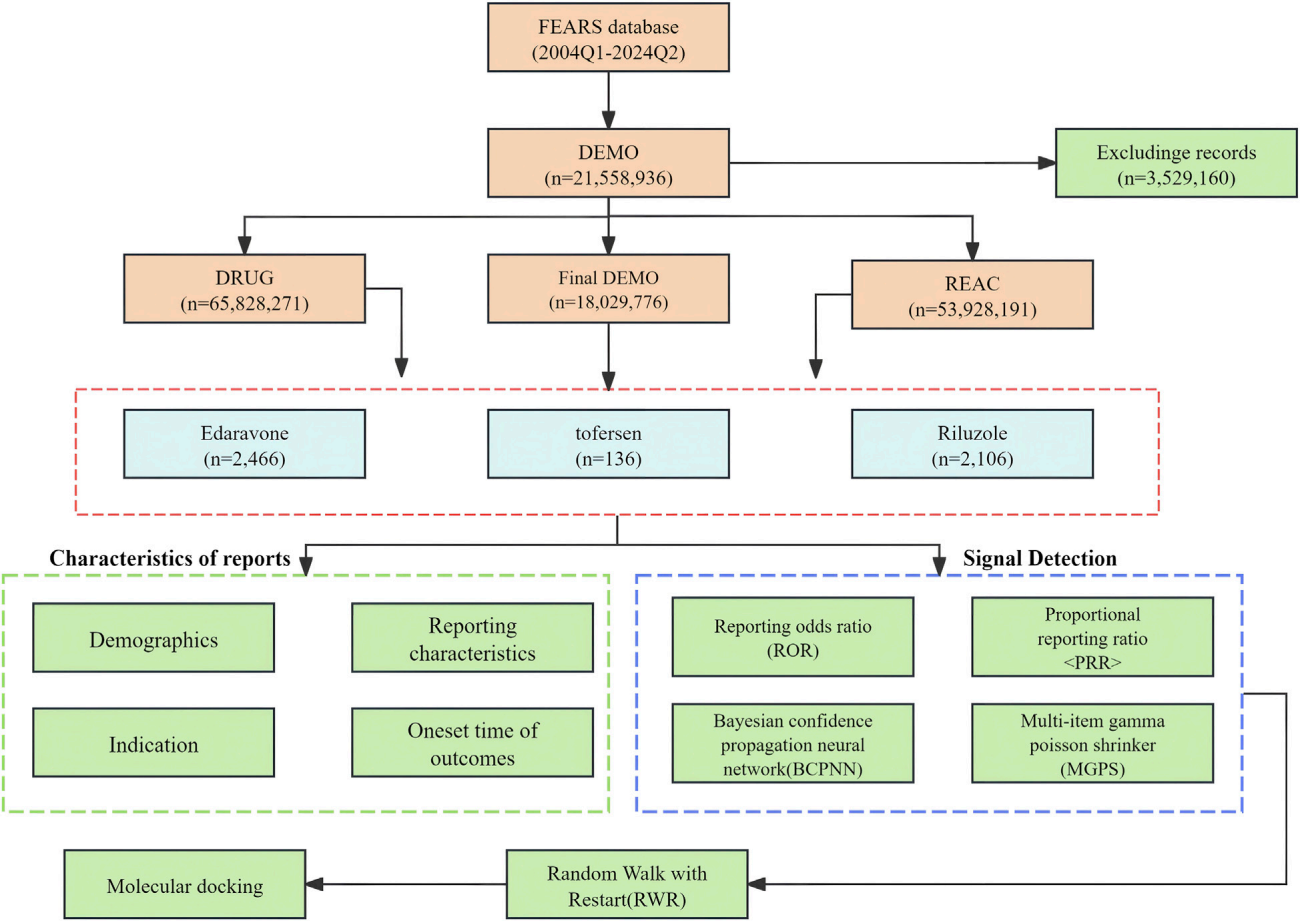
The flow diagram of selecting drugs-related AEs from FAERS database.

**FIGURE 2:**
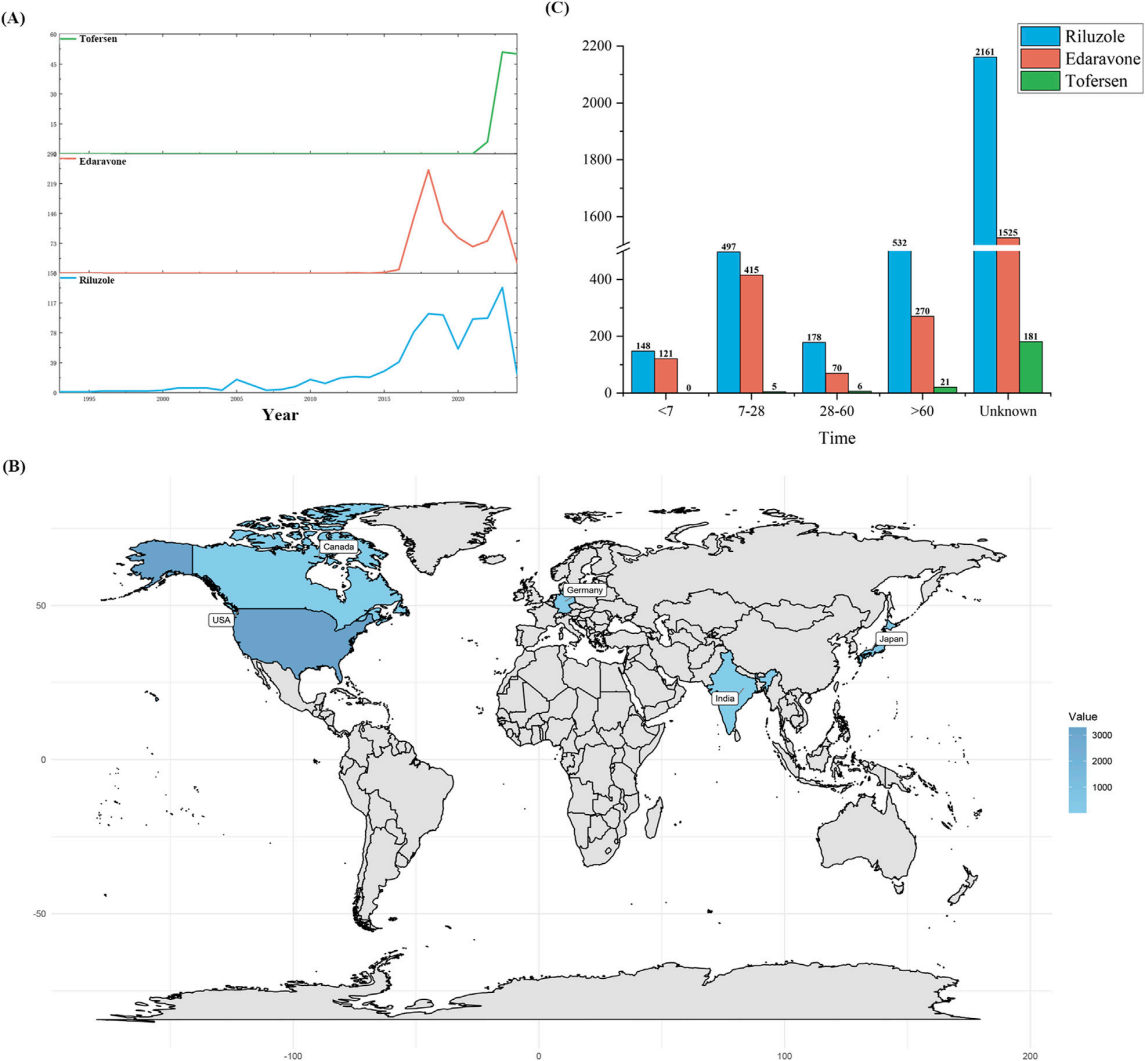
Post-marketing distribution of adverse events for Riluzole, Edaravone, and Tofersen reported on a quarterly basis **(A)** Year **(B)** Global Distribution **(C)** Age distribution.

The extensive dataset compiled by FAERS is depicted in Figure 1, showcasing a total of 21,558,936 AE reports collected from the first quarter of 2004 to the second quarter of 2024. After cleaning the data, FAERS collected a total of 4,708 AE reports, of which 2,106 were for Riluzole, 2,466 for Edaravone, and 136 for Tofersen. The trends in the annual number of ADE reports for Riluzole and Edaravone are roughly the same, both showing a fluctuating increase followed by a decrease from 2015 to 2024. Tofersen began to show an upward trend starting in 2020. Notably, the temporal distribution of these reports reveals a peak in AE reporting for Riluzole and Edaravone in the years 2023 and 2018, with 134 and 252 reports, respectively. Tofersen is a new drug approved for a shorter period of time, whose AE reports do not exist every year but have been accumulated to 136 in the past 3 years, averaging approximately 45 cases per year (Figure 2A). Meanwhile, as shown in Figure 2B, the majority of these reports come from the United States, followed by Germany, as well as other countries such as Japan and Canada. This trend suggests a potential increase in the drug’s utilization or possibly an enhanced vigilance in reporting AEs.

#### 3.1.2 Demographic distribution and reporting sources

Table 3 provides a detailed demographic analysis of AE reports associated with the treatment of ALS using Riluzole, Edaravone, and Tofersen. It indicates a minor majority of male reporters, who account for roughly 44.3% of all reports. Males reported 42.92%, 45.17%, and 49.26% of AE reports for Riluzole, Edaravone, and Tofersen, respectively, indicating a nuanced gender distribution in the reporting pattern. The age distribution trend was largely the same, with the 18–65 age group having the most cases, and the under-18 age group having the fewest patients.

**TABLE 3:** Characteristics of reports associated with Riluzole, Edaravone, and Tofersen extract from Q1 of 2004 to Q2 of 2024.

Characteristic	Riluzole	Edaravone	Tofersen
Gender			
Male	904 (42.92)	1,114 (45.17)	67 (49.26)
Female	748 (35.52)	802 (32.52)	57 (41.91)
Unknown	454 (21.56)	550 (22.30)	12 (8.82)
Age			
0∼18	18 (0.85)	0 (0)	0 (0)
18∼65	728 (34.57)	469 (19.02)	62 (45.59)
≥65	639 (30.34)	494 (20.03)	21 (15.44)
Unknown	721 (34.24)	1,503 (60.95)	53 (38.97)
Reporter			
Consumer	947 (44.97)	1,441 (58.43)	68 (50.00)
Health professional	291 (13.82)	132 (5.35)	18 (13.24)
Lawyer	3 (0.14)	0	0
Pharmacist	381 (18.09)	501 (20.32)	40 (29.41)
Other health professional	262 (12.44)	264 (10.71)	0
Pharmacist	132 (6.27)	125 (5.07)	10 (7.35)
Unknown	90 (4.27)	3 (0.12)	0
Outcomes			
Congenital Anomaly	1 (0.06)	0	0
Death	487 (28.45)	813 (54.49)	18 (24.00)
Disability	35 (2.04)	10 (0.67)	2 (2.67)
Hospitalization initial or prolonged	527 (30.78)	360 (24.13)	33 (44.00)
Life threatening	70 (4.09)	20 (1.34)	2 (2.67)
Other serious (important medical event)	582 (34.00)	284 (19.03)	20 (26.67)
Required intervention to prevent permanent impairment/damage	10 (0.58)	5 (0.34)	0
Country (top 3)			
United States	1,217 (57.79)	1,974 (80.05)	105 (77.21)
United Kingdom	248 (11.78)	0	4 (2.94)
Japan	147 (6.98)	318 (12.90)	0
Canada	0	29 (1.18)	0
Italy	0	0	13 (9.56)

Furthermore, the sources of these AE reports are very similar in the three drugs. For these drugs, consumer reports lead, followed by healthcare physicians, which may reflect a difference in the perceived severity or clinical identification of AEs. This variance in reporting sources is crucial for understanding the pharmacovigilance landscape and is vividly illustrated in Table 3.

#### 3.1.3 Delving into outcome and the temporal dynamics of adverse events

There were 3,279 reports involving serious outcomes, and those related to death accounted for a significant proportion (40.02%). In terms of the proportion of patients who died, Edaravone had the highest percentage (61.68%), while Tofersen had the lowest (1.37%) (Table 3).

The Time-to-Onset (TTO) analysis, depicted in Figure 2C, provides an invaluable perspective on the temporal distribution of AEs. Riluzole and Tofersen exhibited a higher proportion of adverse reactions after 60 days, whereas Edaravone exhibited a significantly higher proportion of reports of AEs occurring within 7–28 days. This temporal aspect of AE reporting offers crucial insights into the onset patterns of adverse reactions, enabling more informed clinical decisions and patient management strategies.

### 3.2 Disproportionality analysis

#### 3.2.1 Analysis of adverse events of Riluzole, Edaravone, and Tofersen

The comprehensive disproportionality analysis of AE reports for Riluzole, Edaravone, and Tofersen, extracted from the FAERS database, reveals significant insights into the safety profiles of these drugs. This analysis, grounded in a robust statistical framework, identified 89, 55, and 15 strong signals.

**FIGURE 3:**
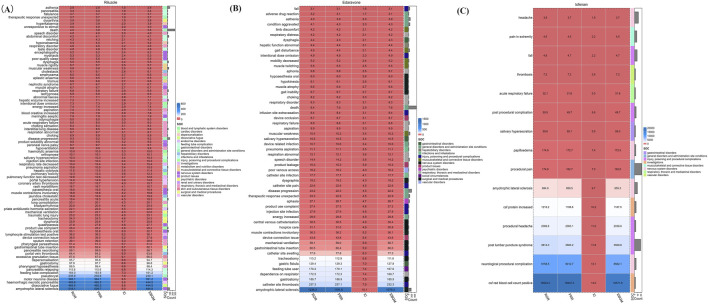
Signal strength of reports of **(A)** Riluzole, **(B)** Edaravone, and **(C)** Tofersen at the at the preferred terms (PT) level in FAERS database.

As shown in Figure 3, these signals, indicative of a statistically significant disproportionality between the observed and expected number of AE reports, highlight potential areas of concern and necessitate a deeper examination of the drugs’ safety profiles. For Riluzole, the analysis delineates a range of AEs specific to its clinical use. Among the notable findings in the SOCs related to general disorders and administration site conditions, death incidents stand out (X^2^ = 615.66, 95% CI lower limit = 3.56, IC-2SD = 1.75). This is closely followed by disease progression (X^2^ = 677.03, 95% CI lower limit = 7.58, IC-2SD = 2.77), and asthenia (X^2^ = 105.34, 95% CI lower limit = 2.29, IC-2SD = 1.14), underscoring critical areas for clinical vigilance. Besides, Riluzole is uniquely associated with abdominal discomfort (X^2^ = 143.41, 95% CI lower limit = 3.24, IC-2SD = 1.60) and hepatic enzyme increased (X^2^ = 215.20, 95% CI lower limit = 5.26, IC-2SD = 2.18) within the gastrointestinal disorders, respectively.

The AE reports of Edaravone were similar to Riluzole. Edaravone exhibits a distinct set of AEs, particularly concentrated within musculoskeletal and connective tissue disorders. Strong signals were identified: gait disturbance (X^2^ = 174.57, 95% CI lower limit = 3.50, IC-2SD = 1.71), mobility decreased (X^2^ = 94.79, 95% CI lower limit = 94.80, IC-2SD = 1.64), limb discomfort (X^2^ = 24.42, 95% CI lower limit = 2.26, IC-2SD = 0.83) and muscle twitching (X^2^ = 37.07, 95% CI lower limit = 2.98, IC-2SD = 1.10), signifying the drug’s pronounced effects on motor function.

The AE reports of Tofersen vary significantly with the other two drugs. Strong signals were identified: procedural pain (X^2^ = 3534.31, 95% CI lower limit = 113.38, IC-2SD = 3.70), post lumbar puncture syndrome (X^2^ = 66650.09, 95% CI lower limit = 2387.44, IC-2SD = 3.63), and headache (X^2^ = 24.32, 95% CI lower limit = 2.16, IC-2SD = 0.79). Understanding the specific AE profiles of these drugs enables clinicians to devise more informed and individualized treatment plans, enhancing patient safety and therapeutic outcomes in the management of ALS.

#### 3.2.2 System disorders analysis of adverse events

**FIGURE 4:**
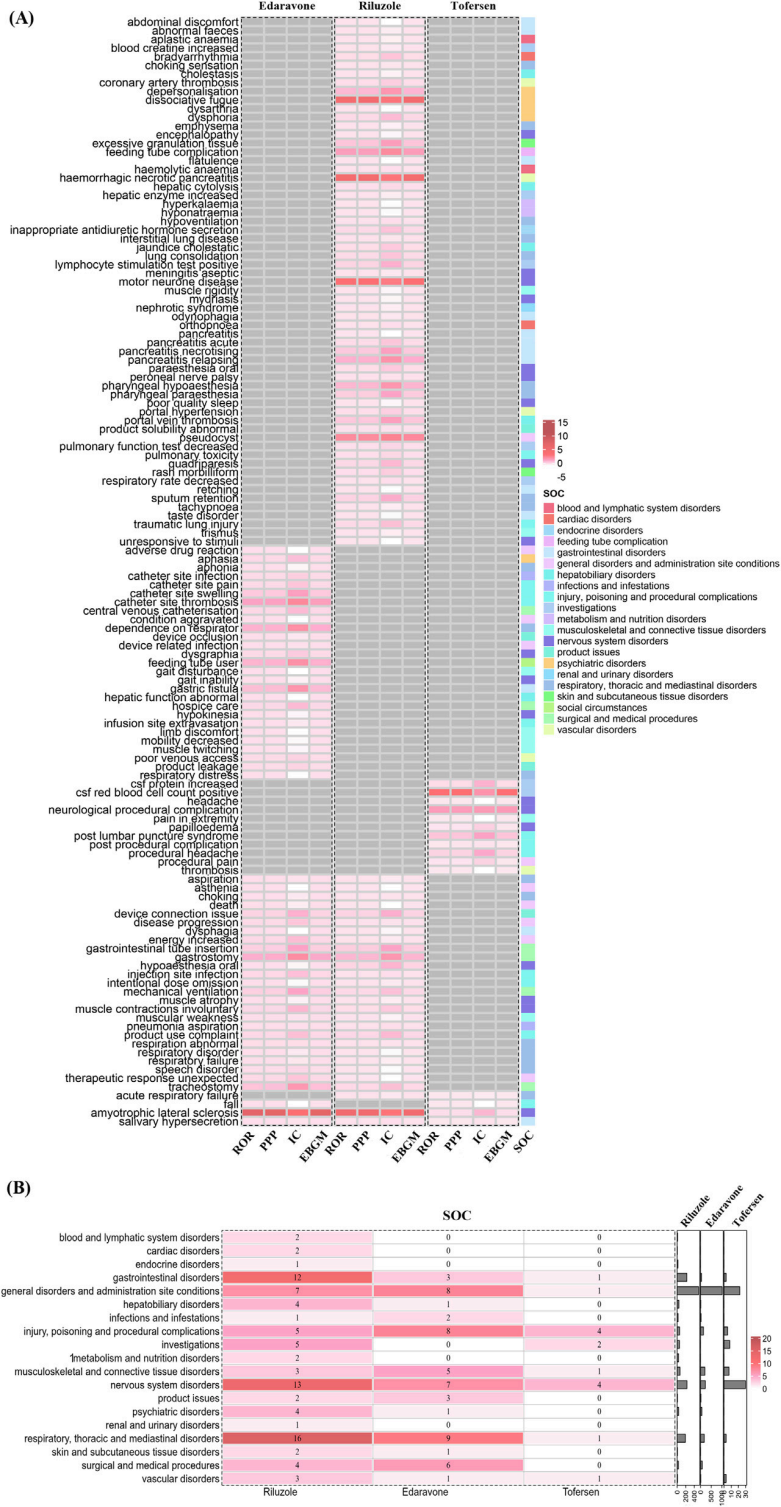
Comprehensive Comparative Analysis of Riluzole, Edaravone, and Tofersen. **(A)** PT level. **(B)** SOC level.

Riluzole’s AEs predominantly affect the general disorders and administration site conditions (499 reports, 6 signals). Following this, nervous system disorders (231 reports, 13 signals), gastrointestinal disorders (227 reports, 12 signals), and respiratory, thoracic and mediastinal disorders (193 reports, 16 signals). Each of these areas presents a distinct facet of Riluzole’s impact on patient wellbeing, underscoring the need for comprehensive monitoring and management strategies to mitigate these risks. Edaravone’s AEs predominantly affect the general disorders and administration site conditions (1,016 reports, 8 signals), nervous system disorders (225 reports, 7 signals), respiratory, thoracic and mediastinal disorders (173 reports, 9 signals), and injury, poisoning and procedural complications (135 reports, 8 signals). Tofersen’s AEs predominantly affect the nervous system disorders (30 reports, 4 signals), investigations (8 reports, 2 signals), and injury, poisoning and procedural complications (5 reports, 4 signals) (Figure 4A).

This distribution provides essential insights into the drug’s varied effects beyond its primary use, highlighting the complexities of managing its side effects.

### 3.3 Comparison of safety signals in system organ classes

Riluzole excavated 89 positive signals involving 20 SOCs. Edaravone found 55 positive signals involving 13 SOCs. Tofersen excavated and found 15 positive signals involving 8 SOCs. Neurological diseases were the SOC shared by three drugs. An in-depth comparison of AE signals across major system organ classes unveiled distinct characteristics for each drug, as detailed in Figure 3. Riluzole and Edaravone emerged with amyotrophic lateral sclerosis in nervous system disorders as the most prominent signal, underscoring a significant concern in its usage. Despite this, the general disorders category for both drugs displayed similar report volumes, indicating some overlap in their AE spectra within this SOC. On the other hand, Tofersen was closely associated with post-lumbar puncture syndrome within injury, poisoning and procedural complications, reflecting its unique safety signal based on ROR and Chi-square analyses.

Comparative analysis of the suspicious positive signals selected by Riluzole, Edaravone, and Tofersen found that the overlapping PTs of the three mainly involved the nervous system. Riluzole and Edaravone overlap 5 PTs: asthenia, death, respiratory failure, muscular weakness, and disease progression. Edaravone and Tofersen have 1 overlapping PT: fall. Riluzole and Tofersen have no overlapping PT (Figure 4A).

#### 3.3.1 In-depth analysis of respiratory and psychiatric disorders adverse events

In the “Respiratory, Thoracic and Mediastinal Disorders” SOC, relevant reports were detected for all three drugs. Riluzole and Edaravone showed a higher number of respiratory system-related reports, corresponding to 16 and 9 signals. In contrast, Tofersen had relatively fewer reports (Figure 4B). From a PT perspective, respiratory failure emerged as an overlapping signal for both Riluzole and Edaravone (Figure 4A), indicating that this PT is relatively common in spontaneous reports associated with these two drugs. In the FAERS database, PTs such as “respiratory insufficiency/respiratory failure,” “dyspnoea,” “hypoventilation,” and “aspiration pneumonia” are often linked to the progressive respiratory muscle weakness inherent to ALS. Additionally, “non-invasive ventilation (NIV)” frequently appears as a medical intervention or procedure-related entry, which may be reported either as an AE or as part of the management strategy due to disease progression.

We also conducted a targeted screening of the “Psychiatric Disorders” SOC and related PTs, including depression, anxiety, insomnia, emotional disorder, irritability, and suicidal ideation. Overall, this study did not identify any “strong and stable” positive signals for psychiatric disorders consistently supported by multiple algorithms across the three drugs. However, sporadic PTs related to mood or sleep disturbances were observed at the spontaneous reporting level. Given the high prevalence of depression, anxiety, and psychological stress in the ALS population, these entries are often affected by issues such as indication confusion and under-reporting, complicating the direct attribution to the drugs. Nonetheless, depression and anxiety may reduce patients expectations and adherence to long-term treatments with modest benefits (such as the life-prolonging effects of Riluzole or the functional slowing effects of Edaravone), potentially impacting medication compliance.

### 3.4 The mechanism of severe adverse reactions

**FIGURE 5:**
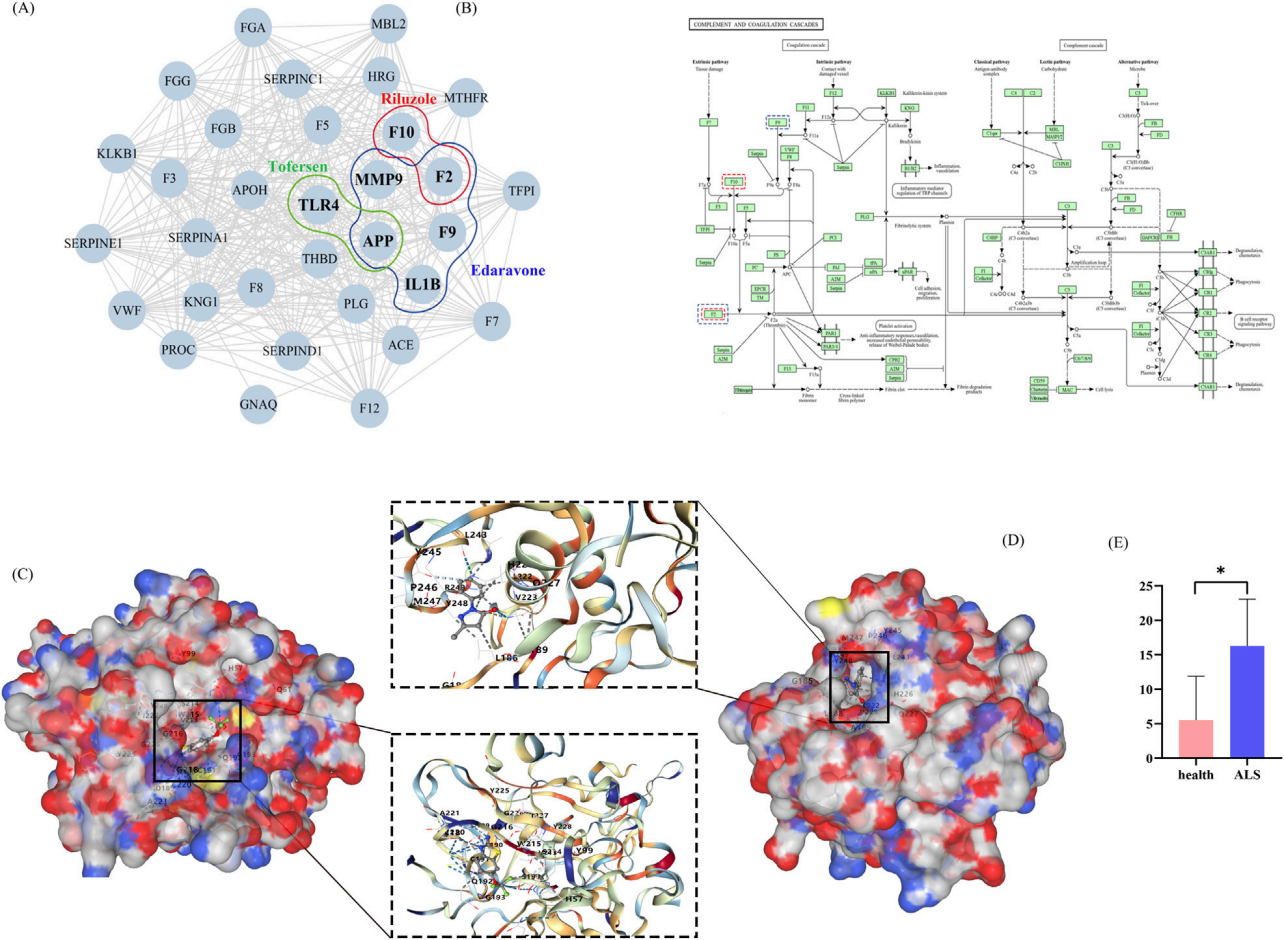
The molecular mechanisms of thrombosis formation caused by Riluzole, Edaravone, and Tofersen. **(A)** The targest network of three drug targets involved in thrombosis. **(B)** Complement and coagulation cascades. **(C)** F10 docking Riluzole. **(D)** MMP9 docking Edaravone. **(E)** Expression of F10 in healthy and ALS groups.

Comparative analysis of three pharmacotherapeutic agents revealed thrombosis as a common adverse event. Through systematic interrogation of biomedical databases (OMIM, HPO, ChEMBL, SwissTargetPrediction), we curated 206 thrombosis-associated genes and 98 pharmacological targets. Network analysis integrating these datasets identified 285 functionally connected proteins within the human interactome. Subsequently, the RWR algorithm was applied to the PPI network, using 10 seed nodes to screen for closely related drug targets (Figure 5A). Its mechanism involves the complement and coagulation cascades (Figure 5B). Utilizing CB-Dock2 to simulate the interaction between the drugs Riluzole and Edaravone with their corresponding gene-encoded proteins, we generated the binding energies of each binding site interaction. The molecular docking results of the protein with the drugs are shown in Figures 5C,D. The binding energy between F10 and Riluzole is −7.8 kcal/mol, and the binding energy between MMP9 and Edaravone is −7.1 kcal/mol, indicating that the binding is very stable. Transcriptome analysis revealed that F10 is highly expressed in ALS patients (*P* < 0.05) (Figure 5E).

## 4 Discussion

This study systematically analyzed the adverse event warnings associated with three medications for the treatment of ALS using the FAERS database. The findings contribute to clinical knowledge by providing comprehensive insights into the types and frequencies of drug-related adverse events. Notably, the age range of 18–65 years, which constituted the majority of reports, aligns with the epidemiological understanding of ALS, thus validating the relevance of our data (Ren et al., 2024). In addition, our analysis revealed that male patients experienced drug-related adverse events more frequently than their female counterparts across all three medications. Given that ALS predominantly affects men, largely due to demographic factors, this observation is consistent with the known male-to-female ratio of approximately 1.5:1 to 2:1 (Marin et al., 2016). This finding, therefore, highlights the importance of gender-conscious management strategies in ALS care.

Moreover, the disproportionality analysis conducted as part of our investigation provided nuanced insights into the adverse event profiles of the three drugs (Miller et al., 2012; Glass and Fournier, 2022). While confirming the known safety profiles, the analysis also underscored the need for vigilance in identifying drugs that may accelerate disease progression, such as those associated with a high incidence of death in ALS patients. In this regard, our findings align with and extend previous clinical studies on Riluzole, Edaravone, and Tofersen, providing a mechanistic framework that clarifies why these ALS therapies are associated with specific AEs.

For instance, the disproportionality analysis identified Riluzole as being linked to fatigue, abdominal discomfort, and elevated liver enzymes. Previous studies have suggested that the primary AEs of Riluzole are predominantly observed in the gastrointestinal and respiratory systems, with common events including fatigue, dizziness, gastrointestinal disturbances, and elevated liver enzyme activity. In a prior study, ALT levels were found to be 2–4 times above normal in 7.8% of cases, while AST levels exceeded normal by fourfold in 14.2% of cases; both enzymes were elevated in 6.5% of the cases. In this study, 6.5% of patients discontinued the drug due to these adverse events, and enzyme levels returned to normal within 2 months of discontinuation of Riluzole (Bensimon et al., 1994). Another study documented ALT elevation in 6.7% of cases and AST elevation in 3.8% (Lacomblez et al., 1996). In addition to liver enzyme alterations, weakness occurred in 8.5% of patients (compared to 7.0% in the placebo group), and nausea was reported in 4.9% of patients (compared to 3.5% in the placebo group) (Bensimon et al., 2002), alongside a higher incidence of pancreatitis (Sun et al., 2023; Lacomblez et al., 1996), which aligns with our findings. Riluzole has been shown to be well-tolerated in clinical settings for up to 7 years or more (Fang et al., 2018; Lacomblez et al., 2002). However, a recent reports indicate that two ALS patients developed recurrent pancreatitis within 3 months of initiating treatment with Riluzole (Falcão de Campos and de Carvalho, 2017). These reports emphasize the importance of vigilant monitoring for adverse events, particularly during the first 6 months of treatment.

Such adverse events are biologically plausible due to Riluzole’s anti-excitotoxic mechanism, which involves glutamatergic modulation that affects voltage-gated sodium channels, as well as potential interactions with non-glutamatergic neurotransmitter systems. Furthermore, its reliance on hepatic biotransformation offers a plausible explanation for the observed elevations in ALT/AST levels and the onset of pancreatitis. Hepatotoxicity remains a critical concern, as elevated liver enzymes in some patients have led to the discontinuation of treatment (Bensimon et al., 1994), underscoring the need for proactive monitoring of liver function tests (LFTs) and the establishment of clear guidelines for treatment re-challenge or cessation. Our analysis also reveals an association, at the signal level, between Riluzole and reports categorized as disease progression. While this association is non-causal in the context of spontaneous reporting, it highlights the importance of differentiating between natural disease progression and drug-related events in clinical practice.

Turning to edaravone, two clinical studies have reported instances of orthostatic dysregulation, gait disturbances, shortness of breath, diarrhea, dyschezia, headache, stomatitis, upper respiratory inflammation, and fatalities in relation to edaravone use. Our analysis corroborates these findings, highlighting the significance of falls and language/speech disorders. These results align with those of the Writing Group & Edaravone (MCI-186) ALS 19 Study Group (2017) and Abe et al. (2014). Furthermore, three randomized, placebo-controlled clinical trials revealed that edaravone was associated with a higher incidence of adverse events (AEs) ≥2%, including dermatitis, contact dermatitis, gait disturbances, and contusions, compared to the placebo. These findings are consistent with our results (Kalin et al., 2017). Mechanistically, edaravone’s role as a free-radical scavenger, targeting oxidative stress, supports its neuroprotective intent. Furthermore, factors such as infusion cycles, autonomic vulnerability in ALS, and potential sedative co-medications provide a non-causal yet coherent explanation for the observed falls and gait disturbances. This insight can inform the development of fall-prevention strategies and monitoring during cycle phases.

Regarding Tofersen, a newer intrathecal antisense oligonucleotide (ASO) targeting SOD1, published adverse event data remain relatively limited. Nevertheless, our analysis is consistent with previous research, which reported instances of post-lumbar puncture syndrome, CSF red blood cell positivity, procedural pain, and headache (Miller et al., 2020). These events can be adequately explained by the route and modality of administration, specifically, CSF pressure fluctuations and leptomeningeal irritation at the CSF–meningeal interface, rather than systemic toxicity. This observation underscores the importance of meticulous lumbar puncture technique, post-lumbar puncture care, and interval scheduling as practical measures to mitigate these adverse effects.

In addition, our study also analyzed the thrombosis target points of three types of drugs. F2, which is considered to be closely related to thrombus formation, showed significant enrichment in the complement and coagulation cascades through pathway enrichment analysis. The activation of the complement system not only enhances the coagulation properties of blood by directly damaging vascular endothelial cells, but also releases anaphylatoxins as indirect procoagulants, promoting thrombosis formation (Conway, 2018; Merle et al., 2015; Wei et al., 2024). Coagulation factor X (F10/FX) is a critical component of the coagulation cascade, where it activates thrombin to facilitate platelet aggregation and clot formation, thereby preventing excessive bleeding. Genetic variants in F10, such as those found in Parazacco spilurus subsp. Spilurus can lead to FX deficiency, impairing coagulation function. Notably, our study observed elevated F10 expression in ALS patients compared to healthy controls, which may contribute to the increased thrombotic risk observed in ALS patients following certain treatments (Menegatti and Peyvandi, 2024). Recent studies have demonstrated that abnormal activation of the immune system, particularly the complement system, is closely associated with the progression of ALS (Feldman et al., 2022). Activation of the complement system can trigger localized inflammatory responses through C3a and C5a receptors, which may exacerbate neuronal damage (Merle et al., 2015). By integrating clinical observations with molecular mechanism research, we have further elucidated the complex relationship between thrombosis, immune activation, and ALS. The co-activation of the complement system and coagulation cascade pathways serves not only as a primary mechanism for thrombosis formation, but may also contribute to the progression of ALS pathology through their effects on neurons and the blood-brain barrier (Festoff and Dockendorff, 2021). Taken together, analysis of adverse drug reaction data reveals that medications such as Riluzole, Edaravone, and Tofersen may induce severe adverse reactions like thrombosis through these pathways, particularly *via* targets like F10 and MMP9, further complicating this relationship.

The novelty of our study lies in its extensive use of the FAERS database, providing three real-world comparative safety analyses. This approach offers a broader and more detailed view of the AE profile than clinical trials alone. Additionally, integrating genetic and biomarker data can enhance the personalization of ALS treatment, improving both efficacy and safety. While drugs such as Riluzole, Edaravone, and Tofersen demonstrate clinical value in ALS treatment, their side effects—including gastrointestinal reactions, liver impairment, and blood clotting—pose significant health risks. These adverse effects can accelerate disease progression, worsen prognosis, and increase mortality. Consequently, ALS treatment requires not only the evaluation of drug efficacy but also a comprehensive assessment of potential side effects, especially in incurable cases. Cost-benefit analyses, tailored to individual patient conditions, should ensure that therapeutic benefits outweigh risks. For example, the association between Riluzole and Edaravone with liver damage and gastrointestinal discomfort underscores the need for enhanced safety monitoring in patients with hepatic dysfunction. Similarly, Tofersen’s thrombosis risk warrants close clinical surveillance during treatment. Despite these side effects, Riluzole, Edaravone, and Tofersen remain essential in ALS management. These medications can slow disease progression and extend survival, making them invaluable in the absence of a cure. However, clinical practice must prioritize personalized treatment approaches that balance therapeutic efficacy with potential risks to ensure patient safety and quality of life. Future research should focus on the long-term impact of these side effects and strengthen drug safety monitoring to optimize treatment strategies and advance personalized ALS therapies.

This study conducted a comprehensive pharmacovigilance analysis using the FAERS database; however, it has several limitations. First, the FAERS database may be subject to biases such as underreporting and variability in report quality. Adverse reactions may be omitted due to delayed patient reporting or incomplete physician documentation, leading to an underestimation of adverse reaction frequency. Additionally, the accuracy and completeness of reports can vary because of the diverse data sources. Second, duplicate reports may occur, especially when multiple sources report the same adverse reactions for one patient, potentially overestimating certain reactions. To mitigate this, we implemented a deduplication process during data cleaning. Regarding Tofersen, its recent approval has led to a limited number of reports in the FAERS database (only 136 reports). This limited data may affect the statistical power of the drug safety analysis, particularly for identifying rare adverse reactions, and restricts our understanding of the long-term safety of Tofersen.

## 5 Conclusion

Our comprehensive analysis emphasizes the indispensable role of pharmacovigilance in optimizing ALS management. As Riluzole, Edaravone, and Tofersen continue to play crucial roles in clinical practice, our study contributes to a deeper understanding of their safety profiles, facilitating informed clinical decision-making and enhancing patient care. Future research, armed with more robust pharmacovigilance methods, will continue to build upon our findings, further advancing the goal of safe and effective ALS treatment.

## Data Availability

The raw data supporting the conclusions of this article will be made available by the authors, without undue reservation.
